# Polyphenols and Cardiometabolic Health: Knowledge and Concern among Romanian People

**DOI:** 10.3390/nu15102281

**Published:** 2023-05-12

**Authors:** Ioana Mariana Haș, Bernadette-Emőke Teleky, Dan-Cristian Vodnar, Bianca Eugenia Ștefănescu, Delia Mirela Tit, Maria Nițescu

**Affiliations:** 1 Doctoral School of Biomedical Sciences, University of Oradea, 410087 Oradea, Romania; ioanahas@gmail.com; 2 Institute of Life Sciences, University of Agricultural Sciences and Veterinary Medicine, 400372 Cluj-Napoca, Romania; bernadette.teleky@usamvcluj.ro (B.-E.T.); dan.vodnar@usamvcluj.ro (D.-C.V.); 3 Department of Food Science and Technology, University of Agricultural Sciences and Veterinary Medicine, 400372 Cluj-Napoca, Romania; 4 Department of Pharmacy, Faculty of Medicine and Pharmacy, University of Oradea, 29 N. Jiga St., 410028 Oradea, Romania; 5 Department of Preclinical–Complementary Sciences, University of Medicine and Pharmacy “Carol Davila”, 050474 Bucharest, Romania; mnitecudsp@gmail.com

**Keywords:** polyphenols, cardiovascular disease, population behavior, education, obesity, awareness

## Abstract

The cardiometabolic health of the population is a crucial indicator of public health, considering the significant impact of cardiovascular disease (CVD) and diabetes on global mortality. Determining the population’s knowledge and the predictors of these pathologies is essential in developing effective educational and clinical strategies for the prevention and management of cardiometabolic risk (CMR). Polyphenols are natural compounds with a multitude of beneficial effects on cardiometabolic health. This study explored the current knowledge, understanding, and awareness of CMR, the benefits of polyphenols among Romanians, and how sociodemographic and clinical characteristics influence this aspect. Five hundred forty-six subjects responded anonymously to an online questionnaire designed to assess their knowledge. The data were collected and analyzed based on gender, age, education level, and BMI status. Most respondents expressed concern to a great or very great extent about their health (78%) and food (60%), with significant differences (*p* < 0.05) depending on age, educational level, and BMI status. Of the respondents, 64.8% declared that they were familiar with the CMR term. Still, the results showed a weak correlation between the stated risk factors and the self-assessment of increased risk (*r* = 0.027) for CVD or diabetes. Only 35% of the respondents reported a good or very good knowledge of the term “polyphenols”, 86% recognized the antioxidant effect, and significantly fewer (26%) recognized the prebiotic effect. Developing and implementing targeted educational strategies to enhance learning and individual behaviors related to CMR factors and the benefits of polyphenols is necessary.

## 1. Introduction

**Figure 1:**
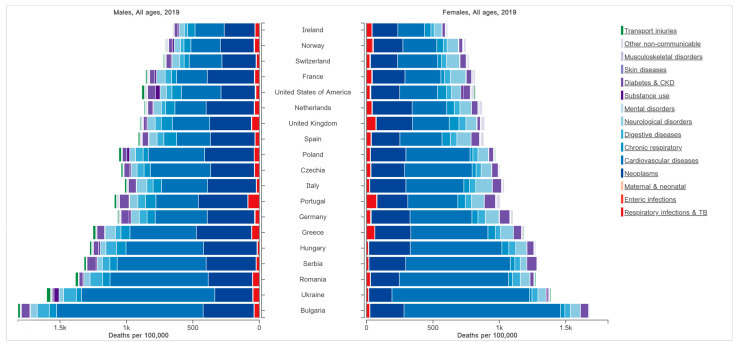
Deaths per 100,000 people in Europe and the United States in 2019 (Adapted with permission from Ref. [3]).

The prevalence of cardiovascular diseases (CVDs) and diabetes are constantly increasing worldwide. According to the World Health Organization (WHO), CVD and diabetes are responsible for most non-communicable disease deaths, totaling 19.5 million people annually [1]. At the same time, we are facing an obesity epidemic worldwide, and this is one of the most critical risk factors for CVD and diabetes [2]. As can be observed in Figure 1, in 2019, CVDs and diabetes were responsible for 56.4% of all deaths in Romania, and Romania is among the first of the countries with the highest death prevalence in Europe [3]. However, at the national level, there are no screening programs for the prevention, early identification, or limitation of the complications that may occur in CVDs and diabetes. A systematic assessment of the cardiometabolic risk (CMR) is necessary. CMR is a broader concept than cardiovascular risk. It refers to the risk of developing CVD, vascular events, and diabetes based on risk factors and metabolic markers (fasting blood glucose, lipid profile, blood pressure, abdominal circumference, age, sex, smoking, and family history) [4].

In developing CVD and diabetes, behavioral risk factors such as unhealthy food consumption, sedentariness, smoking, and excessive alcohol consumption are of significant importance [5]. The role of diet in the prevention of these diseases has been intensively studied [6,7]. In this sense, the most common unhealthy eating habits are considered: a diet high in saturated fat, an increased salt intake, and a low consumption of dietary fiber (cereals, fruits, and vegetables) [8]. A healthy, diversified, balanced diet rich in vegetables and fruits reduces the risks of diabetes and CVD. Fruits and vegetables are excellent sources of bioactive compounds such as polyphenols, which are primarily known for their antioxidant effect [9].

Polyphenols are natural compounds we have at our disposal in fruits, vegetables, whole grains, cocoa, and beverages such as tea or wine [10,11,12]. Polyphenols can be classified as non-flavonoids (phenolic acids, stilbenes, and lignans) and flavonoids (anthocyanins, flavonols, flavones, isoflavones, flavanones, and flavanols) [13,14,15]. Scientific data have shown that the systematic consumption of foods with high contents of polyphenols is correlated with decreasing cardiovascular morbidity and mortality [16], diabetes [17,18], obesity [19], cancer [20], and depression [21]. In terms of cardiometabolic health, polyphenols have been shown to have antioxidant and anti-inflammatory effects [22], antithrombotic effects [23], improve the lipids profile [24] and endothelial function [25], and sustain glucose and insulin homeostasis [26]. More recently, phenolic compounds have also been attributed to the prebiotic effect of beneficial microorganisms, which have been proven to be involved in cardiometabolic health [27,28].

Considering the importance of preventing/treating CMR factors and the benefits of polyphenols in this area, the present study aimed to investigate the current level of knowledge, understanding, and awareness of CMR, as well as the benefits of polyphenols, among Romanians and how sociodemographic and clinical characteristics influence this aspect. The result could help competent stakeholders in the development of screening and educational programs and in the development of novel functional foods or nutraceuticals. As far as we know, this is the first study of this type in Romania.

## 2. Materials and Methods

### 2.1. Online Survey

To research and evaluate the degree of knowledge and consciousness regarding the risk of CVD and the advantages provided by polyphenol consumption among Romanian people, an online, available Google Forms questionnaire was applied [29].

#### 2.1.1. Participants

A total of 546 adult participants living in Romania responded anonymously to an online questionnaire about their knowledge, understanding, and awareness of cardiometabolic risk and the benefits of polyphenols in this regard. Data were collected between November and December 2022. The participants came from urban and rural areas. They were of different ages, sexes, levels of education, weight statuses, smoking statuses, family histories of CVD and diabetes, and clinical characteristics.

#### 2.1.2. Survey Design

As a survey instrument, a three-part questionnaire in Romanian was designed. It consisted of 24 questions, with both open-ended and closed-ended questions (Appendix A). The duration of the questionnaire’s application ranged between 7 and 10 min.

#### 2.1.3. Questionnaire Instrument

The questionnaire consisted of open-ended and closed-ended questions, including rating-scale questions, single-choice questions, multiple-choice questions, and multiple-choice grids. Demographical information such as age, sex, location, and education were required in the first part. Clinical and anthropometric characteristics, such as the presence of elevated blood pressure (or treatment), hyperglycemia (or treatment), elevated low-density lipoprotein cholesterol (LDL-c) or triglyceride (TG) levels, height, and weight, were also inquired about in this first part. The second part contained questions about the participant’s level of interest in health, about when their most recent general medical consultation was, and when their last set of routine tests took place. Afterward, the respondents were asked if they were aware of the concept of CMR, and through another two questions, we assessed their knowledge deeply (“Which of the following conditions do you think are based on CMR?” and “Which of the following factors do you think may increase CMR?”). The last question in this section addressed the participants’ assessment of their risk of developing CVD or diabetes in the next ten years. The third part was focused on their level of interest in food, the type of their diet, and their frequency of consuming certain foods rich in polyphenols such as berries, whole grains, nuts and seeds, onions, garlic, cocoa, dark chocolate, and olive oil. Another two questions assessed their knowledge of the term “polyphenols”, the link between the composition of the food mentioned above and polyphenols, and the role of polyphenols in cardiometabolic health. To assess the respondents’ perception of the parties responsible for informing them about the potential beneficial effects of polyphenols, respondents had six answer options: a doctor, nutritionist/dietitian, pharmacist, school, the online environment, and specialized sites. The last question assessed the respondent’s choice for supplementation: a functional food or a nutraceutical.

#### 2.1.4. Statistics

Data from the results of the questionnaire were analyzed using Microsoft Office Excel Professional Plus 2016. The results are represented as the mean value of the obtained answers ± the standard deviation (SD), with a confidence level of 95% [30]. A one-way ANOVA and a post hoc-Tukey test were employed to assess the differences between the samples. The symbols utilized were ^NS^ *p* > 0.05, * *p* < 0.05, and ** *p* < 0.01. A Chi-square test was conducted using SPSS, Version 19 (SPSS Inc., Chicago, IL, USA), to compare the results obtained from the questionnaire with the expected results.

## 3. Results and Discussions

### 3.1. Respondents’ Characteristics

The sociodemographic and clinical characteristics of the participants are presented in Table 1.

Most respondents were female (80.6%), as observed in several previous online investigations [29,31,32]. This aspect can be assigned to the fact that women are usually more preoccupied with their health status and the health status of their family. Women are also the ones responsible for the purchase and preparation of foodstuffs. Additionally, most respondents were from an urban environment, and most had a higher education level (Bachelor’s, Master’s, or Ph.D.).

The cardiometabolic health status depends intensely on demographic, socio-economic characteristics, and lifestyle, including dietary habits [33]. According to the evidence, the urban area appears to be an essential factor that affects the cardiometabolic health status. On one hand, it affects the cardiometabolic health status in a positive way (easy access to healthcare services, special spaces designed to support an active lifestyle, and better sanitation); on the other hand, it has negative implications (unhealthy eating behavior, sedentariness, exposure to stress, and increased pollution) [34,35]. However, urbanization also penetrates rural areas, reducing physical activity, increasing exposure to the obesogenic environment, and providing easier access to ultra-processed, calorically dense, and low-nutrition foods [36], increasing the prevalence of risk factors for cardiometabolic diseases [37].

Regarding the influence of sex on the prevalence of cardiometabolic diseases, it is known that estrogen has a protective effect on the cardiovascular system [38]. Therefore, in premenopausal women, the incidence of CVD is lower compared to men of the same age. However, things change after menopause, when hormonal changes favor the onset of several diseases [39], including CVD and diabetes. This aspect is crucial for the prevention of CVD and diabetes in clinical practice [40]. Regarding obesity, beyond the fact that it is widespread in both sexes, an increased level of subcutaneous adipose tissue was identified in the case of women. At the same time, men have a higher level of visceral fat [41,42]. In both sexes, visceral fat mass is more strongly associated with CMR [42].

Level of education has an essential impact on health. Several studies have shown that an increased education correlates with a lower CMR. In contrast, a low level of education correlates with poor health and a shorter life expectancy, even in developed countries [43,44]. First, an increased level of education correlates with a better and implicitly higher-level job, which facilitates access to preventive and acute medical care. Later, social and mental resources are associated with the higher capacity of educated persons to seek connections to manage health problems in stressful situations. In addition, people who are more educated pay more attention to their health, develop healthier habits, are more concerned about scientific evidence, and are more adherent to medical services and treatment when it is needed [45,46].

### 3.2. Health and Nutrition Concerns

**Figure 2:**
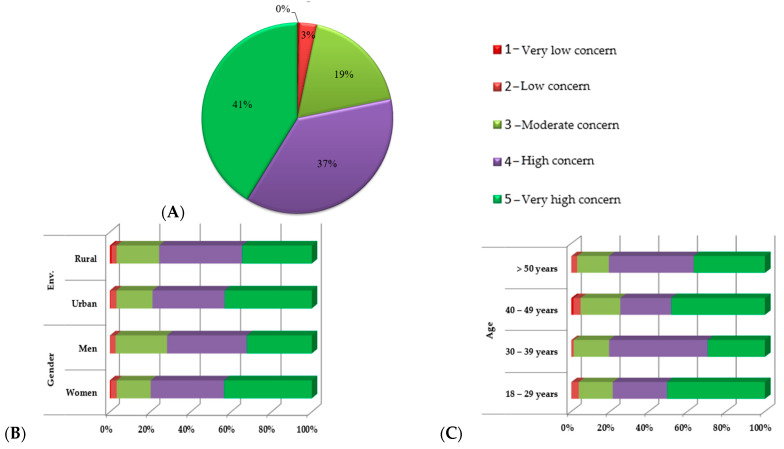

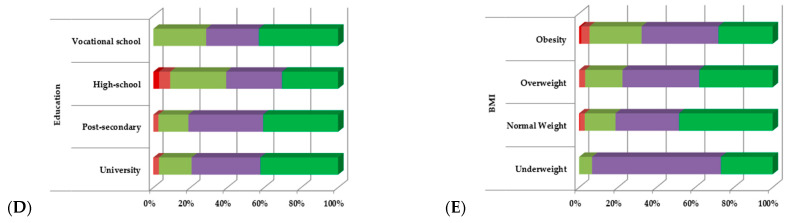
(**A**) Health concern rate among responders, correlated with (**B**) gender, (**C**) age, (**D**) education level, and (**E**) BMI.

The respondents from Romania were grouped based on their gender, age, education level, and BMI status, as shown in Figure 2A–E. When analyzing the degree of concern for health on a scale from 1 to 5 on which 1 represents the lowest degree of interest and 5 the highest (Figure 2A), it can be observed that the majority of subjects 78% declared that they were concerned about their health to a great or very great extent. The highest interest was found in the urban environment and among women (Figure 2B).

Concerning the age group of the respondents (Figure 2C), it was noted that all age groups were concerned about their health status, with those in the 30–39 age group and those over 50 years of age reporting the highest degree of concern about their health, 80.61%, taking into account scale scores of 4 and 5. Using the same principle, depending on the level of education (Figure 2D), it was observed that those who had completed vocational school, post-secondary school, and university studies were the most concerned for their health. Those with a high school education were at the opposite pole, with a much lower degree of concern for their health. When evaluating the degree of concern for health according to weight status (Figure 2E), it was observed that the average weight and underweight respondents had the most significant health concerns. On the opposite pole were the obese and overweight respondents, with a considerable difference between the respondents’ weight status and their degree of concern for their health (*p* < 0.05).

**Figure 3:**
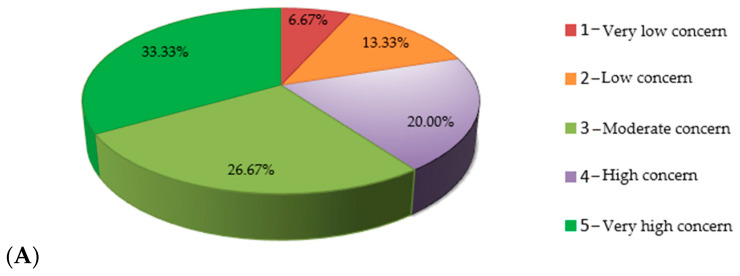

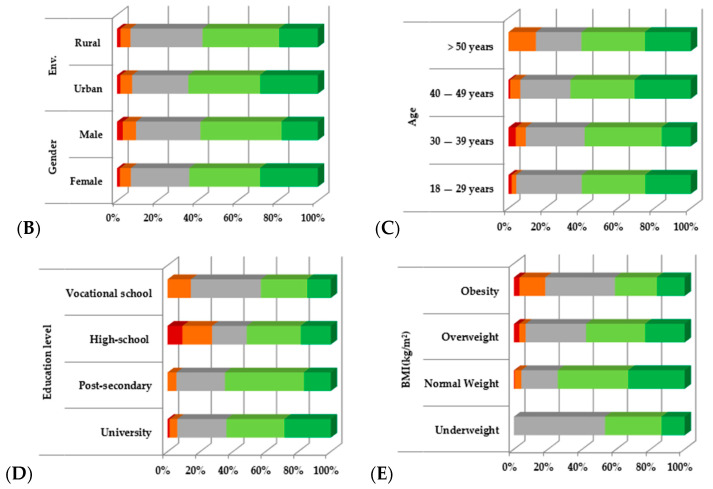
(**A**) Food concerns among participants correlated with (**B**) gender, (**C**) age, (**D**) education level, and (**E**) BMI.

When analyzing the degree of concern for food on a scale from 1 to 5 on which 1 represents the lowest degree of interest and 5 the highest (Figure 3A), it was observed that 60% of the subjects declared that they were concerned about their nutrition to a great or very great extent. The most interest was found in the urban environment and among women (Figure 3B). Regarding the distribution by age categories (Figure 3C), the age category of 40–49 years stood out with the highest degree of concern for food, with a statistically significant difference between the age groups and the degree of interest in food (*p* < 0.05). The respondents’ distribution according to their level of education and concern for their food (Figure 3D) shows that those with a university education were the most concerned about food. At the opposite pole were the respondents with a high school education, with a statistically significant difference between the analyzed variables (*p* < 0.05). Moreover, the most statistically significant difference was noted between the weight status of the respondents and their preoccupation with food (*p* < 0.001) (Figure 3E) in which normal weight subjects had the highest degree of focus, and obese respondents were at the opposite pole.

**Figure 4:**
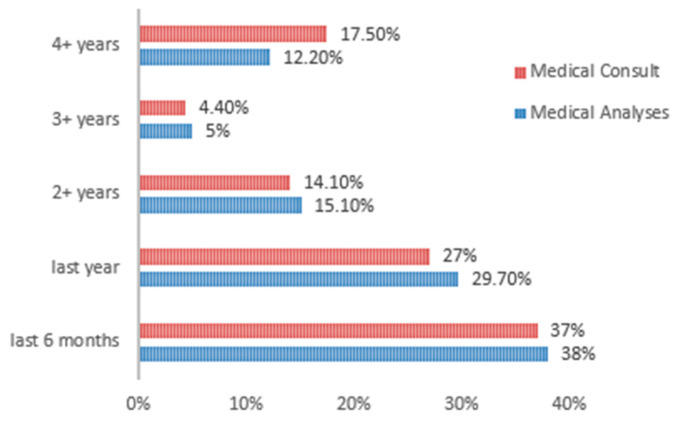
Medical analyses/consults among respondents.

Usual medical analyses were performed more often than a regular medical consultation (Figure 4). Of the respondents, 67.7% declared that they had a set of routine examinations in the last year, and 64% declared that they would have benefited from a medical consultation in the previous year. Those who neglect these aspects, who had medical tests performed or consultations carried out 3 or 4 years ago, were in the proportions of 17.2% and 21.9%, respectively. The data show a moderate correlation between the medical tests and the medical consultation (*r* = 0.55, *p* < 0.001).

According to the WHO definition, health “is a state of complete physical, mental and social well-being and not merely the absence of disease or infirmity” [47], being a fundamental right of every human being [48]. However, inequalities in terms of health will always exist, with socio-economic and genetic differences and personal lifestyle choices being a reality [49]. Food is a factor that significantly influences health, a nutrient-poor diet is one of the risk factors for cardiometabolic diseases [50,51]. Thus, understanding the impact of food choices is crucial in supporting health. However, in people’s minds, nutrition does not always seem to correlate with health. In our study, we found an overlap of increased interest (high and very high) for both health and nutrition in 66.5% of respondents.

The relationship between the level of education, health, and diet has been studied extensively, noting that people with a higher level of education tend to adopt a more nutritious and diverse diet, with a higher consumption of vegetables, fruits, and fish [52,53,54], while diets high in fat and sugar, with an increased share of ultra-processed and nutrient-poor, high-calorie foods, have been associated with a low level of education [54,55,56]. Researchers correlate a higher level of education with the advantage of having the tools to obtain and then correctly understand information about health and the impact of food choices. Moreover, it has been observed that more educated people adopt new aspects faster in any field, including health [54]. The present study also confirms the more significant interest in food among respondents with a higher education level. Beyond the level of education measured in the number of years of schooling, another equally important aspect is the quality of education, which can influence the state of health. Higher levels of health literacy correlate with a better understanding of health services, healthcare strategies, and healthier behaviors [57]. This last aspect is essential in creating short- and long-term strategies for preventing cardiometabolic diseases.

In terms of gender, it is proven that women show a more significant interest than men in terms of health information. They are more attentive and involved in supporting their health and the health of their family, more engaged in providing food, and are generally more attentive to their weight status [58]. This fact was also confirmed in our study, with the concerns for health and nutrition found to be greater among women.

In both cases of concern for health and interest in food, the overweight and obese respondents in our study reported significantly lower levels than the average or underweight respondents. An increased body mass index (BMI) seems to be one of the factors underlying a behavior of avoidance toward healthcare visits [59]. A previous study showed that canceling or postponing doctor’s appointments, in the case of an overweight group, was due to a feeling of embarrassment with respect to their body weight, the fear of being weighed, and the intention to lose weight before going to the doctor to reduce the feeling of stigmatization [60]. This shame is another crucial aspect to consider in designing and implementing various preventive screening interventions precisely because avoiding them would reduce their usefulness. By focusing on health and well-being and on assessing and optimizing habits related to food, physical activity, sleep, and stress and not on directives for weight loss, we could obtain more adherence to screening from this category of people. The involvement of a dietitian in cardiometabolic disease prevention programs could be an effective and practical solution.

Medical controls and laboratory tests are two tools we have at hand to assess health status and prevent diseases. From the data obtained in the present study, correlating the participation in a general medical checkup with the last set of routine tests, it appears that a percentage of almost 20% of those who received their tests did not have a medical checkup. This aspect draws attention to the fact that aside from self-medication, there is also the phenomenon of self-establishing a set of analyses and possibly self-interpreting the results. Laboratory analyses should be based on a medical control; otherwise, based on personal knowledge, or perhaps based on information taken from social media or acquaintances with similar symptoms, there are high chances of missing certain important clinical aspects.

The selective prevention of cardiometabolic diseases aims to identify people at a high risk, who are asymptomatic and without established CMR factors, and then initiate interventions to decrease their CMR [61]. However, some evidence supports that the impact of general health checks on morbidity and mortality is not the desired one [62,63]. Other scientific data show that implementing health checks and targeted interventions decreased the CMR by improving the total cholesterol, BMI, and blood pressure and enhancing the patient’s lifestyle [64,65,66]. As adherence is an essential aspect of a screening program, a recent study tracked the reasons for refusing to participate in a medical checkup aimed at cardiometabolic health. A smoking status, male gender, and the excuse “I think I’m healthy” were most frequently associated with refusing such a control [67].

### 3.3. CMR Awareness and Risk Knowledge

The general knowledge among the respondents regarding the term “Cardiometabolic risk” is represented in Supplemental Figure S1. Of respondents, 64.8% declared they were familiar with the term CMR. The most significant difference regarding the knowledge of this concept was observed in the respondents’ education level. The highest degree of expertise was declared among those with a higher level of education (university and post-secondary education), and the lowest degree of expertise was declared among those with high school education; there is a statistically significant difference (*p* < 0.001) between the level of education and knowledge of the CMR concept. Moreover, this concept was known more by women than by men (*p <* 0.05) and by respondents from urban areas compared to those from rural areas, but with a statistically insignificant difference (*p >* 0.05). Regarding the age groups, the degree of knowledge of the CMR concept was the same. Additionally, in the distribution of respondents according to BMI, we observed that the underweight respondents knew this concept to a greater extent than the obese respondents.

**Figure 5:**
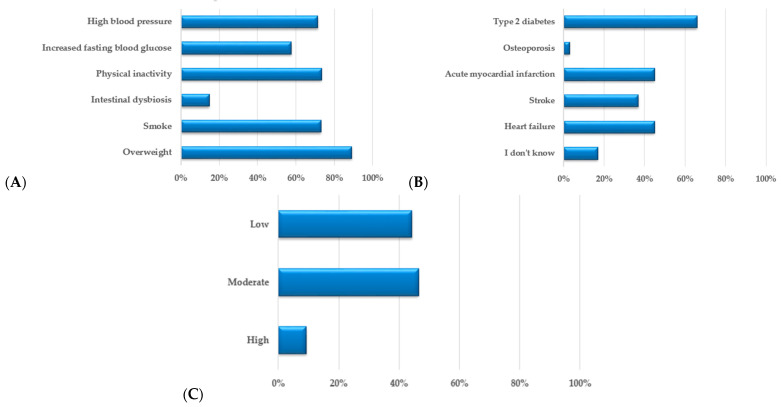
Respondents’ opinions regarding (**A**) factors that increase cardiometabolic risk; (**B**) diseases that are based on cardiometabolic risk; and (**C**) their risk of developing CVD or diabetes in the next 10 years.

As the question “Are you familiar with the term cardiometabolic risk?” does not provide a complete and relevant picture of the respondents’ knowledge, they were asked to point out the factors that may increase the CMR from a list containing six elements, as shown in Figure 5A. Overweight (89.2%), sedentarism (73.5%), smoking (73.2%), and high blood pressure (71.5%) were the risk factors most recognized by the respondents. Of the responders, 17% answered, “I don’t know”. An increased fasting blood glucose concentration, even if it is an essential CMR factor, was known only by 57.7% of responders, while intestinal dysbiosis was known by just 15.0%. To assess their knowledge even more deeply, respondents were asked to choose from a list of conditions related or not to CMR. The diseases associated with CMR are highlighted in Figure 5B. Except for diabetes, which 65.7% of responders selected, the other ailments were recognized by less than half of the participants, more precisely: acute myocardial infarction (44.9%), heart failure (44.3%), and stroke (37.2%).

When asked how they would assess their risk of developing a CVD or diabetes in the next ten years, 9% evaluated it as high, 47% as moderate, and 44% as low, as shown in Figure 5C. The data show a strong correlation between the number of reported risk factors and a self-rating of a low risk (*r* = −0.801); a moderate negative correlation was found between the number of declared risk factors and a personal assessment of moderate risk (*r* = 0.520). However, a weak correlation was observed between the stated risk factors and the self-assessment for an increased risk (*r* = 0.027). Regarding those with more than two CMR factors, 65.9% of those with three risk factors, 58.1% with four risk factors, and 55.6% with six risk factors estimated their risk of developing CVD or diabetes in the next ten years as moderate. Those who were most aware of the danger already had six risk factors; 75% of them evaluated their risk as high, but even here, the remaining 25% assessed their risk as low.

Numerous studies have assessed CVD- or metabolic-syndrome-related knowledge [68,69,70]. However, few have investigated the ability specifically with respect to CMR [71,72]. As far as we know, this was the first study to examine the knowledge about CMR among Romanian adults. CMR refers to the risk of developing CVD, vascular events, and diabetes based on risk factors, with the essential factors being an increased fasting blood glucose concentration, low-density lipoprotein cholesterol or triglyceride levels, high blood pressure, overweight, smoking, alcohol consumption, sedentarism, or stress [73].

Suppose the degree of knowledge of the concept of CMR in the previous studies was either small [71] or relatively high [72]. The current study reflects a reasonably good knowledge of “cardiometabolic risk” among Romanian respondents. Moreover, to a relatively high degree, the respondents identified most risk factors except for increased fasting blood glucose and intestinal dysbiosis.

Patient recognition of CMR factors is definitely the first important step in preventing cardiovascular events and diabetes. However, a question arises here regarding how good the understanding of the impact of risk factors is beyond the ability to identify them. Therefore, it is interesting to emphasize here that even if an essential percentage of respondents identified excessive weight as a risk factor, correlating BMI and the declared risk factors, it appears that 34.2% of overweight and obese people do not realize that they have a weight that is greater than normal. This aspect is pronounced in overweight people, with 48.4% of overweight individuals considering themselves to not be overweight. This aspect is worrying because overweight people may not self-identify as having excess weight and may not be aware of the importance of the various types of intervention and prevention. Therefore, with an adequate understanding of the CMR factors, patients could be mindful of the negative impact of CMR and the importance of prevention in this regard.

Excessive weight, and especially central obesity, is a major CMR factor, exerting its effects even independently of other risk factors, a fact that was revealed in several studies [74,75]. By accumulating excess adipose tissue, the structure and function of the heart change, and in the process of adaptation, an increase in blood and plasma volume and cardiac output occurs [74]. Over time, coronary heart disease develops, and the risk of sudden cardiac death increases. Another mechanism that explains the impact of excess weight on cardiometabolic health is the release of adipocytokines from adipose tissue which induce systemic inflammation, hypercoagulability, endothelial dysfunction, and insulin resistance and will eventually lead to blood vessel damage, atherosclerosis, and diabetes [76].

Cohort studies have shown that high blood pressure (BP) is a significant risk factor for heart failure, atrial fibrillation, coronary heart disease, and stroke [77,78]. A medical condition characterized by too much blood pressure in the blood vessels, which, if not controlled, can affect the heart, kidneys, and brain [77]. According to a WHO estimation, 46% of hypertensive adults are unaware that they have this condition [79]. According to the WHO, diabetes is a major cause of myocardial infarction, stroke, kidney failure, blindness, and lower limb amputation [80]. Increased glucose levels can directly affect endothelial function, increase oxidative stress, activate the polyol pathway, and determine the evolution of atherosclerosis or heart failure [81].

It is well known that regular physical activity is closely correlated with reducing cardiovascular risk, decreasing insulin resistance, and improving body composition, among other health benefits [82]. In particular, previous studies showed that physical activities such as aerobics, cycling, and lifting weights can lead to a decrease in blood pressure and improvements in the lipid profile, blood sugar, and body weight, aspects that support the primary and secondary prevention of cardiovascular events in both adolescents and adults [82,83].

Smoking is another important risk factor, and it has been proven that both in active and passive forms, it increases the risk of developing cardiovascular and metabolic diseases. Among the harmful effects of smoking are the atherogenic and prothrombotic effects, the alteration of the lipid metabolism, tissue lipotoxicity, and insulin resistance [84,85].

Numerous pieces of evidence point out the involvement of the intestinal microbiome in the development and progression of various diseases, including CVDs and type 2 diabetes, due to intestinal dysbiosis [86,87]. The mechanisms researched and emphasized in this direction are inflammation production, epithelial barrier dysfunction, and energy metabolism dysregulation [88]. Even if access to all this information is effortless these days, it must be transmitted in a simplified and easy-to-understand form, regardless of the level of education or field of training.

Under the CVD umbrella is a group of disorders of the heart and blood vessels. According to statistics, 85% of mortality cases with cardiovascular causes were due to heart attack and stroke [89], and heart failure is one of the most prevalent CVDs [90]. For this reason, we chose these three diseases as answer options for the question about conditions related to CMR. Unfortunately, below-average knowledge was registered for myocardial infarction, heart failure, and stroke as CMR-related conditions, a sign that upon a more thorough investigation, the degree of knowledge and understanding is no longer as good as the degree of “familiarity” of the concept of CMR.

Another important aspect is the respondents’ perception of the risk of developing a CVD or diabetes in the next ten years. According to previous data, there is often a tendency to underestimate the risk of coronary heart disease among patients [91], an aspect also confirmed in our study. It was also previously observed that women have a greater perception of severity regarding various risk factors [91]. In this case, this was not confirmed; the perception of women was similar to that of men when we referred to those with at least three CMR factors present. Additionally, even if, in agreement with the previous findings [70,92], the people with a higher level of education in the current study had a higher degree of knowledge about CMR, the perception of their own CMR was low. Finally, 75% of people with at least three risk factors assessed their risk of developing CVD or diabetes as low or moderate.

### 3.4. Polyphenol Knowledge

#### 3.4.1. Consumption Frequency

As can be seen in the table below (Table 2), herbs or aromatic spices (161 ± 3.1%) and onions, garlic (142 ± 1.8%), and olive oil (129 ± 2.9%) present the largest share of daily consumption. Likewise, whole grains (180 ± 2.2%), cocoa powder, and dark chocolate (176 ± 1.1%) are consumed more often weekly than daily. The food products consumed more often than once a month are represented by berries (161 ± 1.4%), nuts (155 ± 1.5%), and seeds (137 ± 1.1%). At the opposite pole, seeds (86 ± 0.7%) stand out for representing the highest share of consumption in the category of less-frequent consumption, being consumed around once or twice per year or never.

#### 3.4.2. General Knowledge of the Term “Polyphenols”

**Figure 6:**
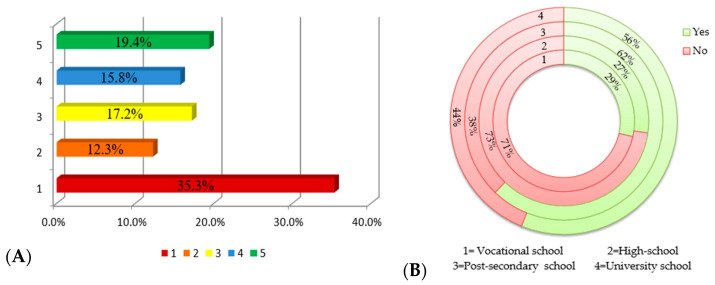
(**A**) Respondents’ knowledge of the term “polyphenols”; (**B**) identification of polyphenol sources based on education.

As can be seen in Figure 6A, for almost half of the respondents, precisely 47.6%, the term “polyphenols” was totally unknown or slightly familiar, and only 19.4% of the respondents know this term very well. Afterwards, when asked if they knew that the foods for which they had just provided information regarding their frequency of consumption are sources of polyphenols, i.e., natural compounds with a multitude of beneficial effects on cardiometabolic health, 45.6% of the subjects answered no. The highest level of knowledge was registered among respondents with a post-secondary education (62%) and university education (56%), with the difference between them and the respondents with vocational school and high school education being extremely statistically significant (*p <* 0.001).

#### 3.4.3. Health-Related Aspects of “Polyphenols”

Regarding the respondents’ opinions regarding the benefits of polyphenols for cardiometabolic health, 86% recognized the antioxidant effect, 57% the glycemic control activity, 54% the anti-inflammatory effect, and only half of the study participants identified the lipid-profile control effect. Fewer participants recognized the prebiotic effect (26%) and the effect of improving endothelial function (25%).

**Figure 7:**
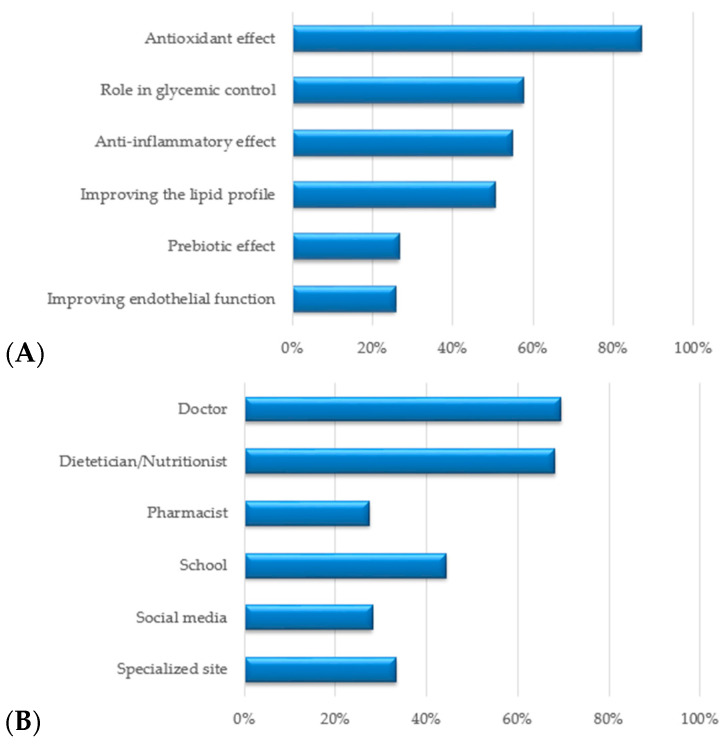
(**A**) Cardiometabolic health benefits of polyphenols based on respondents’ opinions; (**B**) information sources regarding the role of polyphenols.

Finally, regarding the perception of the responsibility to inform the population about polyphenols and their beneficial effects on cardiometabolic health, the percentages are more than eloquent: 69% and 68% thought that doctors and dietitians/nutritionists should be responsible for information, respectively, while 44% of participants thought that children should be informed at school as part of health and nutrition education courses, and 33% would prefer to receive this information from specialized sites, 28% from social media, and 27% from pharmacists. (Figure 7B).

Evidence shows that an unhealthy diet and other modifiable risk factors play a significant role in developing chronic, non-communicable diseases. Up to 80% of CVDs could be prevented if these risk factors were eliminated [93].

In recent years, polyphenols have gained significant research interest precisely because of their multiple beneficial effects. An increasing amount of scientific evidence correlates the regular consumption of foods rich in phenolic compounds with a lower CMR [94,95,96]. However, the current study highlights a low level of knowledge of the term “polyphenols”, with only 35% of the respondents reporting a good or very good understanding of it.

The study data showed that most respondents, 78.5%, declared that they had an omnivorous diet, and 17.4% claimed that they followed a flexitarian diet. Much lower percentages were recorded in the case of participants who were vegetarians (3.4%) and vegans (0.7%). Regarding the consumption of foods containing phenolic compounds, among the respondents of the current study, the highest frequency was found in spices, onions, garlic, and olive oil. At the same time, berries, cocoa, seeds and nuts, and integral cereals, showed lower frequencies of consumption. According to Eurostat data, in 2019, the most insufficient daily intake of fruits and vegetables in the European Union was found in Romania, where “only 2% of the population ate at least five portions of fruit and vegetables” [97]. In the respondents’ responses, when choosing between a functional food or a food supplement if they needed to supplement their intake of polyphenols, 77.7% opted for available functional food.

Berry fruits have been intensively studied in recent decades for their beneficial effects on human health as an excellent source of polyphenols, especially anthocyanins. In addition to anthocyanins, they contain phenolic acids, flavonols, and stilbenes. The highest amounts of phenolic compounds are usually found in the skin of berries [98]. Cocoa beans are also a rich source of phenolic compounds, mainly flavonols, flavan-3-ols, and anthocyanins [99]. Nuts and seeds are notable for their phenolic acids, flavonols, flavan-3-ols, flavanones, stilbenes, and lignans [100,101], and whole grains are especially noted for their content of phenolic acids and also flavonoids [10]. The consumption of all these foods was correlated with a cardiometabolic protective effect.

The beneficial effects of polyphenols are due to their antioxidant capacity, ability to induce the synthesis of detoxification enzymes, and the ability modulate intra- and intercellular signaling pathways [102,103,104]. Polyphenols protect the endothelium, reduce cholesterol levels and LDL oxidation [105], decrease the synthesis of pro-inflammatory cytokines and adhesion molecules, play an essential role in glucoregulation, and reduce insulin resistance [13,106].

A more recently studied aspect is the prebiotic potential of phenolic compounds. Intestinal dysbiosis is associated with the development and progression several non-communicable diseases, including CVDs, diabetes, and obesity [107,108,109]. In vitro and in vivo studies highlighted the bidirectional relationship between polyphenols and the intestinal microbiota. More precisely, phenolic compounds can modulate the intestinal microbiota and participate in the host’s intestinal balance. In contrast, the activity of polyphenols is modulated by microorganisms, such as their structure and secondary metabolites [13,108]. A summary of the main benefits of polyphenols for cardiometabolic health, in addition to their classification and dietary sources, is presented in Supplemental Table S1.

According to the results of our study, antioxidant activity is known among the population, while the prebiotic potential is almost unknown. Even if the demand for prebiotics and probiotics is constantly increasing at the global level [29,110], our awareness of natural sources of prebiotics must be improved. This aspect can be due to a need for more information or an adaptation to the level of understanding of the general population.

## 4. Conclusions

The current study reflects reasonably good knowledge of the term “cardiometabolic risk” among Romanian respondents. Moreover, to a relatively high degree, the respondents identified most risk factors except for increased fasting blood glucose level and intestinal dysbiosis. Nevertheless, a weak correlation between the stated risk factors and the self-assessment of increased risk (*r* = 0.027) for CVD or diabetes was found, suggesting the need to deepen public knowledge with respect to recognizing risk factors and the correct awareness of the risk of developing CVD or diabetes.

Even if a high percentage of respondents reported a heightened concern for their health and nutrition, the results indicated a deficient level of knowledge about polyphenols and their bioactive effects. With adequate educational programs, many Romanians are provided with information about the benefits of including polyphenols in their daily diet to prevent or control risk factors associated with cardiometabolic pathologies.

Based on the observed results, it is therefore essential for medical professionals to provide the population with correct and evidence-based information in an easy-to-understand manner regarding the health impact of consuming foods rich in. Doctors and dieticians are the most reliable professionals in this regard, detaching significantly from the other sources of information proposed in this study.

Future studies to determine the factors associated with the degree of knowledge and the barriers to communicating information to the population are necessary to indicate specific and effective educational interventions.

## Figures and Tables

**Table 1:** Respondents’ characteristics.

Demographics	*n*	%		*n*	%
Gender			Elevated BP/treatment		
Female	440	80.6	Yes	68	12.5
Male	106	19.4	No	478	87.5
**Provenience**			**Smoking**		
Urban	426	78	Yes	128	23.4
Rural	120	22	No	418	76.6
**Age**			**BMI (kg/m^2^)**		
18–29 years	116	21.2	Underweight	15	2.7
30–39 years	127	23.3	Normal weight	277	50.7
40–49 years	217	39.7	Overweight	161	29.5
>50 years	86	15.8	Obese	93	17
**Education level**			**FH of CVD or Diabetes**		
University	469	85.9	Yes	394	72.2
Post-secondary	37	6.8	No	131	24
High school	33	6	Don’t know	21	3.9
Vocational school	7	1.3			
**Hyperglycemia/treatment**			**Elevated LDL-c**		
Yes	20	3.7	Yes	85	15.6
No	526	96.3	No	461	84.4
**Elevated TG**					
Yes	42	7.7			
No	504	923			

BMI—body mass index, FH—family history, CVD—cardiovascular disease, *n*—number of respondents, TG—triglyceride, BP—blood pressure, LDL-c—low-density lipoprotein cholesterol.

**Table 2:** Consumption frequency of food products.

Food Products	Consumption Frequency (%)					
	Daily	Once/ Week	Several Times/ Week	Several Times/ Month	1–2 Times/ Year	Not at All
Whole grains	52 ± 1.3 ^b^	123 ± 2.2 ^b^	180 ± 2.4 ^b^	99 ± 1.6 ^c^	52 ± 1.3 ^b^	40 ± 1.1 ^a^
Berries	26 ± 1.8 ^c^	120 ± 2.8 ^b^	159 ± 1.2 ^c^	161 ± 2.1 ^a^	69 ± 1.5 ^b^	11 ± 0.9 ^b^
Nuts	39 ± 2.1 ^c^	133 ± 1.7 ^a^	150 ± 1.6 ^c^	155 ± 2.3 ^a^	58 ± 1.2 ^b^	11 ± 1.0 ^b^
Seeds	25 ± 1.2 ^c^	121 ± 3.3 ^b^	130 ± 2.7 ^d^	137 ± 2.7 ^b^	86 ± 1.7 ^a^	47 ± 1.4 ^a^
Aromatic herbs/spices	161 ± 3.1 ^a^	82 ± 1.5 ^c^	185 ± 3.0 ^b^	64 ± 1.5 ^d^	27 ± 0.9 ^c^	27 ± 1.3 ^a^
Cocoa powder/dark chocolate	17 ± 1.0 ^c^	155 ± 3.7 ^a^	176 ± 3.1 ^b^	117 ± 2.3 ^b^	55 ± 1.3 ^b^	26 ± 1.5 ^a^
Olive oil	129 ± 2.9 ^a^	93 ± 1.4 ^c^	191 ± 2.3 ^b^	64 ± 1.4 ^d^	37 ± 1.2 ^c^	32 ± 1.1 ^a^
Onion/Garlic	142 ± 1.8 ^a^	95 ± 1.7 ^c^	249 ± 2.7 ^a^	38 ± 1.2 ^e^	14 ± 0.8 ^c^	8 ± 0.5 ^b^

Respondent results are presented as mean values ± SD in each column. The significant differences (*p* < 0.05) are shown with distinct superscript letters (a–e) within the types of food products.

## Data Availability

Data are contained within the article.

## Supplementary Materials

The following supporting information can be downloaded at: https://www.mdpi.com/article/10.3390/nu15102281/s1, Figure S1: Awareness and knowledge regarding the term “Cardiometabolic risk”; Table S1: Main benefits, classification, and dietary sources of polyphenols in cardiometabolic health. References [111,112,113,114,115,116,117,118,119,120,121,122,123,124,125,126,127,128,129] are cited in the supplementary materials.

## Appendix A. Questionnaire

Cardiometabolic risk and the benefits of polyphenols—level of knowledge and understanding among Romanians.
