# Supplementary material for: Polyphenols and Cardiometabolic Health: Knowledge and Concern among Romanian People
Source: Nutrients. 2023 May 12;15(10):2281. doi: 10.3390/nu15102281 (PMC10221773; doi:10.3390/nu15102281)
Supplement: Supplementary file 1 [file nutrients-15-02281-s001.zip › nutrients-2396786-supplementary.pdf]

Figure S1. Awareness and knowledge regarding the term "Cardiometabolic risk"

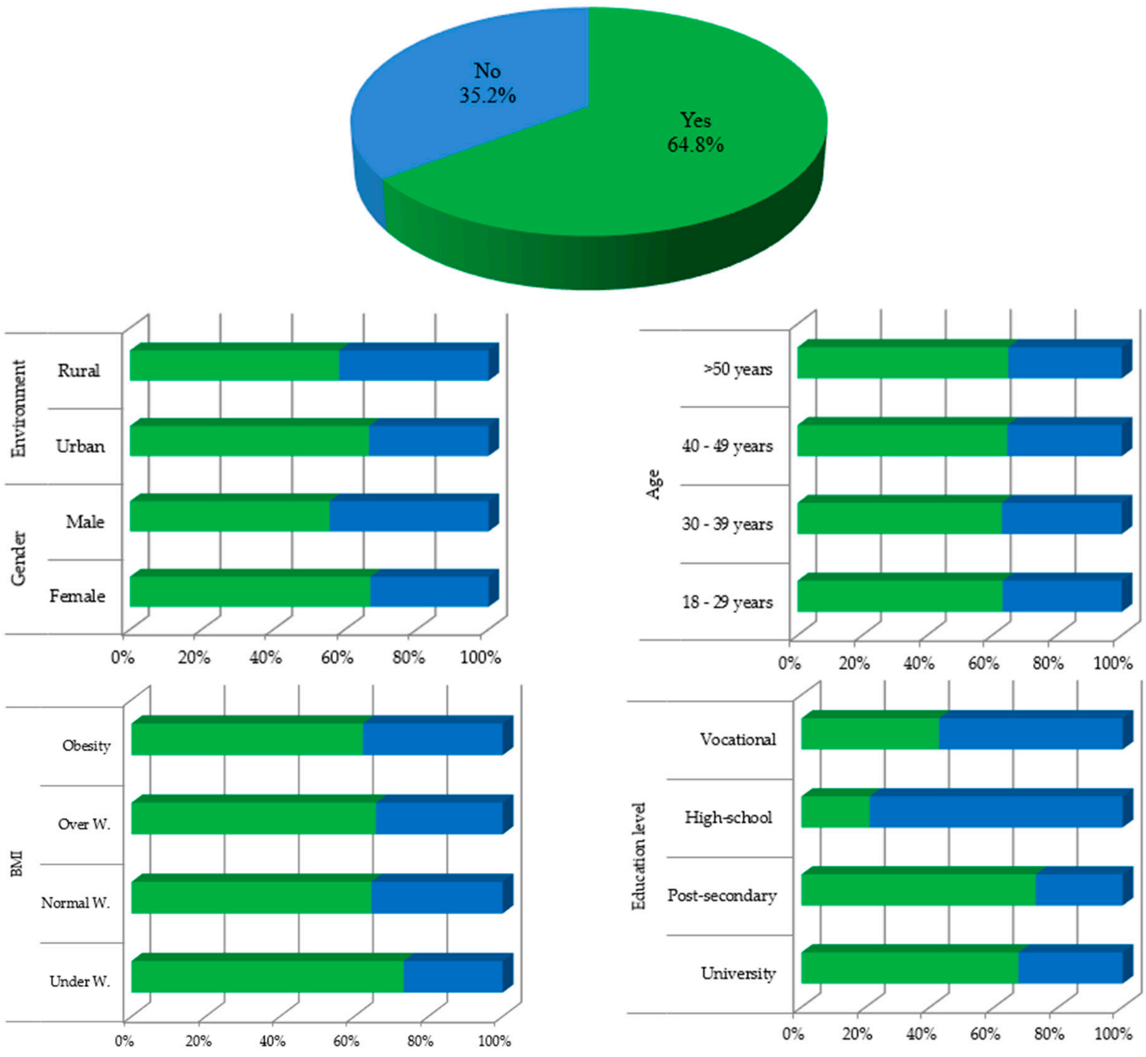

Table S1. Main benefits, classification, and dietary sources of polyphenols in cardiometabolic health

Class	Subclass	Compounds (examples)	Main dietary sources	Main benefits	Ref.
Flavonoids	Flavonols	Quercetin, kaempferol, myricetin	apple, berries, leeks, onions, broccoli; beans, kale, tea, red wine, tomato	**Antioxidant activity** (by capturing reactive oxygen and nitrogen species, inhibiting pro-oxidant enzyme and redox-sensitive transcription factors, activating antioxidant enzymes, metal chelator potential)	[1–3]
	Flavones	Tangeretin, luteolin, nobiletin, apigenin, sinsensetin	skin of the citrus fruits, celery, parsley, grains, vegetable oils	**Improves lipidic profile** (by reduction in TC, LDL-C and TG, lowering LDL oxidation)	[2,4–6]
	Isoflavones	Genistein, daidzein, glycitein	Soy and soy products, legumes	**Improves vascular health and blood pressure** (stimulates endothelial NO synthase, reduce arterial stiffness, decrease systolic and diastolic BP)	[7–10]
	Flavanones	Naringenin, eridicytol, hesperetin	Citrus fruits , tomatoes		
	Flavanols	Catechins, Epicatechins, epigallocatechins	Berries fruits, apple red onions, eggplant, nuts, black and green tea, red grapes, red wine, cocoa or dark chocolate	**Anti-platelet activity** (inhibit platelet hyperactivity, reduce platelet aggregation)	[11,12]
	Anthocyanins	Cyanidin, delphinidin, malvidin, petunidin, peonidin	Berries fruits, oranges, mango, olives, red onions, cabbage, beans, red wine	**Protection against atherosclerotic plaque development or growth** (decreases inflammation, decreases the expression of adhesion molecules, decreases the potential of macrophages to oxidize LDL)	[13,14]

Class	Subclass	Compounds (examples)	Main dietary sources	Main benefits	Ref.
Phenolic acids	Hydroxy cinnamic acids	*p*-hydroxy cinnamic acid, cinnamic acid, caffeic acid, ferulic acid, *p*-coumaric acid, sinapic acid	Whole grains, coffee, red wine, vegetables, red fruits	**Improves beta cells activity and insulin action** (reduce markers of inflamation, protect β cells agains oxidative stress results, inhibit β cells apoptosis, protection of beta cells from glucose toxicity, normalizes production and secretion of insulin)	[15,16]
	Benzoic acid derivatives	*p*-hydroxybenzoic acid, gallic acid, protocatechuic acid, ellagic acid	Berries fruits, spices, grains	**Decreases intestinal absorption of dietary carbohydrate** (by inhibiting α-amylase, α-glucosidase, SGLT1 and SGLT2)	[17,18]
Stilbenes		resveratrol, viniferins	Grapes, red wine	**Adjustment of carbohydrate metabolism** (stimulates glycogenesis and glycolysis, decrease gluconeogenesis and glucose output of the liver)	[1,19]
Lignans		Secoisolariciresinol, matairesinol, pinoresinol, sesamin	Flaxseed, sesame seed, whole grains, olive oil, legumes, vegetables, fruits	**Improves cardiometabolic status trought polyphenol-gut microbiota interactions** (promotes the growth of beneficial bacteria, modulates intestinal barrier function, reduces low-grade systemic inflammation by decreasing the influx of LPS into the circulation, modulate gastrointestinal metabolic pathways)	[20,21]

TC- total cholesterol, LDL-C- Low-density lipoprotein cholesterol, TG- triglycerides, NO- nitric oxide, BP- blood presure, SGLT1 and SGLT2- sodium-glucose linked transporters

References

1. Rudrapal, M.; Khairnar, S.J.; Khan, J.; Dukhyil, A. Bin; Ansari, M.A.; Alomary, M.N.; Alshabrmi, F.M.; Palai, S.; Deb, P.K.; Devi, R. Dietary Polyphenols and Their Role in Oxidative Stress-Induced Human Diseases: Insights Into Protective Effects, Antioxidant Potentials and Mechanism(s) of Action. *Front. Pharmacol.* **2022**, *13*, 1–15, doi:10.3389/fphar.2022.806470.
2. Hussain, H.; Green, I.R. A patent review of the therapeutic potential of isoflavones (2012-2016). *Expert Opin. Ther. Pat.* **2017**, *27*, 1135–1146, doi:10.1080/13543776.2017.1339791.
3. Kunnumakkara, A.B.; Bordoloi, D.; Padmavathi, G.; Monisha, J.; Roy, N.K.; Prasad, S.; Aggarwal, B.B. Curcumin, the golden nutraceutical: multitargeting for multiple chronic diseases. *Br. J. Pharmacol.* **2017**, *174*, 1325–1348, doi:10.1111/bph.13621.
4. Panahi, Y.; Kianpour, P.; Mohtashami, R.; Jafari, R.; Simental-Mendiá, L.E.; Sahebkar, A. Curcumin Lowers Serum Lipids and Uric Acid in Subjects with Nonalcoholic Fatty Liver Disease: A Randomized Controlled Trial. *J. Cardiovasc. Pharmacol.* **2016**, *68*, 223–229, doi:10.1097/FJC.0000000000000406.
5. Kubota, S.; Tanaka, Y.; Nagaoka, S. Ellagic acid affects mRNA expression levels of genes that regulate cholesterol metabolism in HepG2 cells. *Biosci. Biotechnol. Biochem.* **2019**, *83*, 952–959, doi:10.1080/09168451.2019.1576498.
6. Amarowicz, R. Natural phenolic compounds protect LDL against oxidation. *Eur. J. Lipid Sci. Technol.* **2016**, *118*, 677–679, doi:10.1002/ejlt.201600077.
7. Serreli, G.; Deiana, M. Role of Dietary Polyphenols in the Activity and Expression of Nitric Oxide Synthases: A Review. *Antioxidants* **2023**, *12*, doi:10.3390/antiox12010147.
8. Furuuchi, R.; Shimizu, I.; Yoshida, Y.; Hayashi, Y.; Ikegami, R.; Minamino, T. Boysenberry polyphenol inhibits endothelial dysfunction and improves vascular health. **2018**, 1–16.
9. De Bruyne, T.; Steenput, B.; Roth, L.; De Meyer, G.R.Y.; Dos Santos, C.N.; Valentová, K.; Dambrova, M.; Hermans, N. Dietary polyphenols targeting arterial stiffness: Interplay of contributing mechanisms and gut microbiome-related Metabolism. *Nutrients* 2019, *11*, 1–43.
10. Behl, T.; Bungau, S.; Kumar, K.; Zengin, G.; Khan, F.; Kumar, A.; Kaur, R.; Venkatachalam, T.; Tit, D.M.; Vesa, C.M.; et al. Pleotropic Effects of Polyphenols in Cardiovascular System. *Biomed. Pharmacother.* **2020**, *130*, 110714, doi:10.1016/j.biopha.2020.110714.
11. Stainer, A.R.; Sasikumar, P.; Bye, A.P.; Unsworth, A.J.; Holbrook, L.M.; Tindall, M.; Lovegrove, J.A.; Gibbins, J.M. The Metabolites of the Dietary Flavonoid Quercetin Possess Potent Antithrombotic Activity , and Interact with Aspirin to Enhance Antiplatelet Effects. **2019**, 244–258.
12. Ludovici, V.; Barthelmes, J.; Nägele, M.P.; Flammer, A.J.; Sudano, I. Polyphenols: Anti-Platelet Nutraceutical? **2018**, 146–157, doi:10.2174/1381612823666171109104600.
13. Kim, Y.; Cho, A.Y.; Kim, H.C.; Ryu, D.; Jo, S.A.; Jung, Y. Effects of Natural Polyphenols on Oxidative Stress-Mediated Blood-Brain Barrier Dysfunction. **2022**, 1–25.
14. Ahmadi, A.; Jamialahmadi, T.; Sahebkar, A. Polyphenols and atherosclerosis : A critical review of clinical effects on LDL oxidation. *Pharmacol. Res.* **2022**, *184*, 106414, doi:10.1016/j.phrs.2022.106414.
15. Sun, C.; Zhao, C.; Guven, E.C.; Simal-gandara, J.; Ramkumar, K.M.; Buleu, F.; Tomas, M.; Paoli, P.; Wang, S.; Pah, A.; et al. Dietary polyphenols as antidiabetic agents : Advances and opportunities. **2020**, 1–27, doi:10.1002/fft2.15.
16. Williamson, G.; Sheedy, K. Effects of polyphenols on insulin resistance. *Nutrients* 2020, *12*, 1–19.
17. Sun, L.; Miao, M. Dietary polyphenols modulate starch digestion and glycaemic level : a review. *Crit. Rev. Food Sci. Nutr.* **2019**, *0*, 1–15, doi:10.1080/10408398.2018.1544883.
18. Williamson, G. Effects of Polyphenols on Glucose-Induced Metabolic Changes in Healthy Human Subjects and on Glucose Transporters. **2022**, *2101113*, 1–10, doi:10.1002/mnfr.202101113.
19. Golovinskaia, O.; Wang, C.K. The hypoglycemic potential of phenolics from functional foods and

their mechanisms. *Food Sci. Hum. Wellness* 2023, *12*, 986–1007.
20. Villa-rodriguez, J.A.; Ifie, I.; Gonzalez-aguilar, G.A.; Roopchand, D.E. The Gastrointestinal Tract as Prime Site for Cardiometabolic Protection by Dietary Polyphenols. **2019**, 1–13.
21. Fraga, C.G.; Croft, K.D.; Kennedy, D.O.; Tomás-Barberán, F.A. The effects of polyphenols and other bioactives on human health. *Food Funct.* **2019**, *10*, 514–528, doi:10.1039/c8fo01997e.
